# Contribution of different pneumococcal virulence factors to experimental meningitis in mice

**DOI:** 10.1186/1471-2334-13-444

**Published:** 2013-09-24

**Authors:** Susanna Ricci, Alice Gerlini, Andrea Pammolli, Damiana Chiavolini, Velia Braione, Sergio Antonio Tripodi, Bruna Colombari, Elisabetta Blasi, Marco Rinaldo Oggioni, Samuele Peppoloni, Gianni Pozzi

**Affiliations:** 1 Department of Medical Biotechnologies, Laboratory of Molecular Microbiology and Biotechnology (LA.M.M.B.), University of Siena and Siena University Hospital, Siena 53100, Italy; 2 Department of Physiopathology, Experimental Medicine and Public Health, University of Siena, Siena, Italy; 3 Department of Pathology, Siena University Hospital, Siena, Italy; 4 Department of Diagnostics, Clinical and Public Health Medicine, University of Modena and Reggio Emilia (Unimore), Emilia-Romagna, Italy; 5 Present address: Evans Medical Research Center, Boston University School of Medicine, 650 Albany Street, Boston, MA 02118, USA; 6 Present address: Sanofi-Aventis S.p.a., Brindisi 72100, Italy

**Keywords:** Experimental pneumococcal meningitis, Microglia, PspA, PspC, Capsule

## Abstract

**Background:**

Pneumococcal meningitis (PM) is a life-threatening disease with a high case-fatality rate and elevated risk for serious neurological sequelae. In this study, we investigated the contribution of three major virulence factors of *Streptococcus pneumoniae*, the capsule, pneumococcal surface protein A (PspA) and C (PspC), to the pathogenesis of experimental PM.

**Methods:**

Mice were challenged by the intracranial route with the serotype 4 TIGR4 strain (wt) and three isogenic mutants devoid of PspA, PspC, and the capsule. Survival, bacterial counts, and brain histology were carried out. To study the interaction between *S. pneumoniae* mutants and microglia, phagocytosis and survival experiments were performed using the BV2 mouse microglial cell line.

**Results:**

Virulence of the PspC mutant was comparable to that of TIGR4. In contrast, survival of animals challenged with the PspA mutant was significantly increased compared with the wt, and the mutant was also impaired at replicating in the brain and blood of infected mice. Brain histology indicated that all strains, except for the unencapsulated mutant, caused PM. Analysis of inflammation and damage in the brain of mice infected with TIGR4 or its unencapsulated mutant demonstrated that the rough strain was unable to induce inflammation and neuronal injury, even at high challenge doses. Results with BV2 cells showed no differences in phagocytic uptake between wt and mutants. In survival assays, however, the PspA mutant showed significantly reduced survival in microglia compared with the wt.

**Conclusions:**

PspA contributed to PM pathogenesis possibly by interacting with microglia at early infection stages, while PspC had limited importance in the disease. The rough mutant did not cause brain inflammation, neuronal damage or mouse death, strengthening the key role of the capsule in PM.

## Background

*Streptococcus pneumoniae* is a coloniser of the human nasopharynx and can also cause other diseases, including sinusitis, otitis media, pneumonia, sepsis and meningitis. The microorganism produces a plethora of virulence factors, including the polysaccharide capsule, several surface-located proteins, and the toxin pneumolysin [1,2]. The capsule is a major virulence determinant due to its anti-phagocytic activity [3-5]. Among the surface-associated proteins, the pneumococcal surface protein A (PspA) and C (PspC) are the best characterised choline-binding proteins. PspA interferes with complement activation and deposition mediated by both the classical and alternative pathways [6-9] and also binds lactoferrin [10]. PspC interacts with human immunoglobin A and with the polymeric immunoglobulin receptor [11,12], thereby promoting adhesion and transcytosis of pneumococci across mucosal surfaces [13,14]. PspC also shows anti-phagocytic properties due to its capability to bind to complement C3 [15] and factor H [11,16-18].

Pneumococcal meningitis (PM) is a life-threatening disease with high rates of mortality and neurological sequelae [19,20]. The hallmark of meningitis is represented by cerebrospinal fluid (CSF) pleocytosis, which largely contributes to brain inflammation and damage [20,21]. It is well accepted that meningitis-induced brain injury depends on both the host inflammatory response and the direct bacterial toxicity [19-21]. The pneumococcal cell wall and pneumolysin initiate immune activation in the CSF by engaging the toll-like receptors 2 and 4, respectively [22]. Peptidoglycan and teichoic acid have been shown to trigger meningeal inflammation in experimental PM [23,24]. Pneumolysin interferes with the beat frequency of brain ependymal cilia [25], damages the blood-brain-barrier (BBB) [26], mediates apoptosis of microglial and neuronal cells *in vitro*[27] and participates in hearing loss and cochlear damage associated with experimental PM [28]; moreover, a pneumolysin-deficient mutant showed reduced virulence in murine PM [29]. Other pneumococcal knock-out mutants have been analysed in PM models, including strains devoid of the neuroaminidases NanA and NanB [29], the hyaluronidase [29] and the fibronectin-binding protein PavA [30]. To our knowledge, so far the role of PspA and PspC has not been assessed in experimental PM.

Microglial cells, comprising about 15% of brain cells, are located within the brain parenchyma and constitute the main phagocytic population of the central nervous system (CNS) [31,32]. Activated microglia can wield several effector functions, such as phagocytosis, inflammatory responses and antigen presentation [32]. During infection of the CNS, microglial cells secrete pro-inflammatory mediators involved in the recruitment of peripheral immune cells to the site of infection and also exert antimicrobial activity towards invading pathogens [33]. Microglia is therefore considered a key player in the initial innate immune response against CNS infections.

In the present study, we tested the serotype 4 TIGR4 strain and three isogenic mutants deficient in PspA, PspC and capsule in an intracranic mouse model of PM. We also focused on anti-phagocytic pneumococcal virulence determinants and on microglial cells, key effectors of innate immunity and first line of defence against *S. pneumoniae* invading the brain, especially in the early phases of PM.

## Methods

### Bacterial strains and growth conditions

*S. pneumoniae* TIGR4 (type 4) and the isogenic mutants FP23 (rough), FP28 (PspC-) and FP262 (PspA-) were used in this work. Bacteria were grown in Tryptic Soy Broth (TSB, Becton Dickinson, Milano, Italy) until mid-logarithmic phase and stored at −80°C with 10% glycerol. Solid media were prepared by addition of 1.5% agar and 3% defibrinated horse blood (Oxoid, Hampshire, UK) to TSB. Counts of colony forming units (cfu) were performed on blood-agar plates at 37°C with 5% CO_2_. When necessary, chloramphenicol, erythromycin and kanamycin were used at the concentrations of 2.5 μg/ml, 1 μg/ml and 500 μg/ml, respectively.

### Construction of knock-out mutants

All mutant strains were generated by gene SOEing [34]. Construction of the unencapsulated derivative of TIGR4 (FP23) and of the PspC-deficient mutant (FP28) has already been described [35,36]. To construct the PspA-deficient strain, the *pspA* gene was replaced with an erythromycin-resistance cassette (*ermB*) [37] using primers IF188 (5′-AAGTGATTTGTGATTGTTGATG-3′) and IF189 (5′-ACCTCTTTAGCTCCTTGGAAG-3′) [38]. Primer pairs employed to amplify the regions upstream (845 bp) and downstream (587 bp) of the *pspA* gene were IF215 (5′-TTGGGCAGTAGTGAGAACTG-3′)/ IF216 (5′-CATCAACAATCACAAATCACTTCAGACTATACTTATATTAAG-3′) and IF217 (5′-CTTCCAAGGAGCTAAAGAGGTGCCGATTAAATTAAAGCATG-3′)/ IF218 (5′-ATCTTCGGTCGCCGTACAGA-3′), respectively. A 2571 bp-long PCR fragment was used to transform TIGR4, and an erythromycin-resistance mutant was selected and designated as strain FP262. Mutant construction was verified by PCR and sequencing.

### Mice, model of meningitis and experimental design

Outbred 8 to 10-week-old female MF1 mice (Harlan Nossan, Monza, Italy) were used. Animal experimentation was approved by the local ethical committee, and all experiments were performed according to institutional and national guidelines (‘Ministero della Salute’ , Decreto no. 72/2012-B). The method to induce PM in mice has been previously reported [39]. Briefly, mice were lightly anesthetised by intraperitoneal (i.p.) injection of xylazine hydrochloride (Bio 98 S.r.l., Bologna, Italy) and zolazepam tiletamine (Virbac S.r.l., Milano, Italy) and inoculated by the intracranial route (i.c.) with 50 μl of the bacterial inoculum using a micro-syringe with 26 gauge needles (Hamilton, Bonaduz, Switzerland). Studies on survival and cfu determinations in tissues were conducted on animal groups infected with 10^2^, 10^3^ and 10^4^ cfu/mouse. For the rough strain FP23, rodents were also infected with larger doses up to 10^7^ cfu/mouse. Animal group sizes are provided in Additional file 1: Tables S1, S2 and S3. Mice were monitored twice a day for clinical signs as described by Sandgren *et al.*[40]. Briefly, disease severity was graded using end-points on a scale of 0–5, with 0 = normal, 1 = piloerection and decreased spontaneous activity, 2 = hunched position and loss of vigilance, 3 = turns upright in > 5 sec when positioned on the back, 4 = does not turn upright, 5 = moribund. Mice were euthanised if/when they reached a score of 4. Body weight and temperature were recorded once per day for 10 days and compared to those of naïve uninfected control mice. Survival was recorded for 10 days. Assessment of PM by histology was carried out on the brain of animals (n = 3/group) infected with 10^4^ cfu/mouse of *S. pneumoniae* strains and sacrificed 48 h post-infection. For histological evaluation of the role of capsule in PM, two groups of mice were infected with 10^5^ cfu/mouse of TIGR4 (n = 6) or FP23 (n = 16) and sacrificed after 24 and 48 h (3–8 mice/time point). To strengthen the results obtained with the dose of 10^5^ cfu, another group of mice (n = 4) was challenged with 10^7^ cfu of FP23 and sacrificed 72 h post-infection. Control mice were inoculated with 50 μl of phosphate buffered saline (PBS).

### Sample collection

Blood and brain were collected from infected mice for histological analysis and cfu counts. Blood samples were obtained by the sub-mandibular vein. For cfu counts, 100 U/ml of heparin (MS Pharma, Milano, Italy) were added to blood samples to prevent coagulation, whereas brains were homogenised in 1 ml of TSB. Blood and brain samples were frozen at −80°C with 10% glycerol until use. Bacterial counts were performed by plating 10-fold dilutions onto blood-agar plates.

### Brain histology

For histological analysis, brains were immediately fixed in formalin for 24 h and then embedded in paraffin according to standard procedures. The brains were entirely sectioned along a coronal plane. Sections were stained with haematoxylin-eosin according to standard techniques. The presence and degree of inflammation and neuronal damage were evaluated by using routine light microscopy (at least 100 power fields were examined). Inflammation was estimated by counting the number of polymorphonuclear cells (PMN) in four different brain regions: superficial meningeal regions over the convexities, frontal interhemispheric region, hippocampal fissure and third ventricle. Based on the number of PMN for each power field, a score was attributed as follows: 0 (0 PMN), 1 (<10 PMN), 2 (10–50 PMN) and 3 (>50 PMN). For every animal, scores of each brain region were summed up into a final inflammation score (IS). Neuronal damage was evaluated by estimating the percent of damaged neurons in one power field and calculating a score as follows: 0 (no damaged neurons), 1 (<10%), 2 (10-30%) and 3 (>30%). Both apoptotic and necrotic neurons were considered injured. Apoptosis was represented by cell shrinkage, homogenous chromatin condensation, nuclear shrinkage and nuclear transformation into apoptotic bodies. Cell swelling, eosinophilic degeneration of the cytoplasm, nuclear shrinkage with chromatin clumping were considered signs of necrosis. Four different brain areas were analysed: neocortex, striatum, hippocampus/dentate gyrus and cerebellum. Scores of each brain region for each mouse were summed, and the resulting number represented the final damage score (DS).

### Microglial cells

The murine microglial cell line BV2 [41] was maintained in RPMI 1640 medium supplemented with 10% heat-inactivated fetal calf serum (hiFCS) (Defined Hyclone, Logan, UT, USA), gentamicin (50 μg/ml; Bio Whittaker, Verviers, Belgium) and l-glutamine (2 mM; EuroClone, Milan, Italy) (complete medium). Cells were detached biweekly by vigorous shaking, and fresh cultures were started at a concentration of 5 × 10^5^/ml.

### Phagocytosis assay

For all fluorescence-based assays, pneumococci were thawed, washed and suspended at the desired concentrations. Staining of bacteria was performed by incubating 10^8^ cfu/ml with 5 mM of Hoechst 33342 (Sigma-Aldrich, St. Louis, MO, USA) in the dark at 37°C for 1 h as described [42,43]. After labelling, pneumococci were washed four times with PBS and then suspended at the desired concentration in complete RPMI medium without antibiotics. To strengthen the attachment of BV2 cells to wells, Lab-Tek II chamber slides (Nalge Nunc International, Naperville, IL, USA) were pretreated with poly-L-lysine (Sigma-Aldrich; 10 μg/well) for 30 min and then washed with PBS. BV2 cells (10^6^/ml, 100 μl/well) were seeded, incubated for 15 min and infected (moi = 10) with 100 μl of 10^7^/ml cfu of Hoechst 33342-labelled *S. pneumoniae* in RPMI containing l-glutamine and normal FCS (nFCS). After incubation for 3 h, cells were treated with trypan blue for 5 min to quench the fluorescence of bound bacteria, washed with PBS to remove extracellular bacteria and fixed for 30 min with 4% paraformaldehyde (PFA) (Sigma-Aldrich) in PBS. Finally, BV2 cells were washed with PBS and treated with ProLong Gold Antifade Reagent (Molecular Probes, Invitrogen, St. Louis, Mo, USA) to suppress the photobleaching effect and preserve the signals of fluorescent molecules. Remaining fluorescence of phagocytosed bacteria was visualised by epifluorescence microscopy. At least 200 microglial cells from each sample were examined, and the percentage of cells with intracellular bacteria was defined as the ratio of the number of BV2 cells containing one or more bacteria to the total number of cells examined.

### Phagolysosome acidification assay

Visualisation of bacteria-containing acidic phagosomes was performed as described [42,43]. Briefly, BV2 cells were infected for 3 h (see phagocytosis assay), washed to eliminate extracellular bacteria, and exposed to 4 μl of the acidotropic dye LysoTracker Red DND-99 (Molecular Probes, Invitrogen) at a final concentration of 5 μM. Thirty minutes before the end of incubation, an additional volume (4 μl) of the same dye was added. Finally, 5 min before the end of incubation, 100 μl of trypan blue were dispensed into each well. After PFA fixing, BV2 cells were washed and treated with ProLong Gold Antifade Reagent (Molecular Probes, Invitrogen). Acidification of phagosomes containing Hoechst 33342-labelled bacteria was visualised by epifluorescence microscopy by the simultaneous appearance of LysoTracker Red DND-99 (red) and Hoechst 33342 (blue) fluorescence within the phagosomes, resulting in purple fluorescence when merging images. For quantitative analysis, the number of bacteria-containing acidic phagosomes per image was determined by counting the number of purple phagosomes within phagocytic cells. The percentage of colocalisation was then calculated as the number of cells with bacteria-containing acidic phagosomes over the total number of phagocytic cells.

### Epifluorescence microscopy

Prior to visualisation, Lab-Tek II chamber slides were washed with PBS and treated with Prolong Gold antifade Reagent (Molecular Probes, Invitrogen). Epifluorescence and differential interference contrast (DIC) microscopy were performed using a Nikon Eclipse 90i imaging system equipped with Nomarski DIC optics (Nikon Instruments Inc., Melville, NY, USA). Samples were photographed with a DS-2Mv Nikon digital camera, and the resulting photographs were analysed by using the Nikon NIS-ELEMENTS version D3.1 software.

### Intracellular survival assay

Bacterial survival inside microglial cells was assessed by performing an antibiotic-protection assay as previously described [42,43]. Briefly, BV2 cells (10^6^/ml) were incubated for 3 h with bacteria (moi = 10) in RPMI with nFCS. Cells were washed with PBS to remove extracellular bacteria and exposed for 1.5 h to gentamicin (150 μg/ml) and vancomycin (10 μg/ml) in RPMI with l-glutamine. BV2 cells were washed twice with PBS and suspended in complete RPMI without antibiotics (time 0). Following 4 h of incubation (time 4), cells were lysed with 0.2% (v/v) Triton X-100 for 15 sec to release intracellular bacteria, and serial dilutions of the lysates were plated onto blood-agar plates. After 36-48 h cfu were counted, and the survival index (SI) of each strain was calculated as the number of cfu at time 4 h divided by the number of cfu at time 0 h. In all assays where microglial BV2 cells were exposed to *S. pneumoniae*, the viability of infected cells was tested and found comparable to that of uninfected control cells.

### Statistical analyses

Detailed data on mouse survival are described in Additional file 1: Table S1 which reports the median survival time (h) with 95% confidence interval (CI) for each mouse group together with the related statistical analysis (Log Rank test). Additional file 1: Tables S2 and S3 describe the mean and standard deviation (SD) of log cfu counts in the brain and blood of infected mice, respectively. Complete data on phagocytosis and intracellular survival of *S. pneumoniae* in BV2 microglial cells are reported (mean ± SD) in Additional file 1: Table S4. Differences between TIGR4 and the mutants in Additional file 1: Tables S2, S3 and S4 were analysed by the Bootstrap (BCa method) performed on 1000 stratified resampling [44]. Data on IS and DS from mice challenged with TIGR4 and FP23 are shown as median with interquartile range (IQR). Analysis of differences in DS and IS was carried out by the Mann–Whitney U test (Table 1). *P* values < 0.05 were considered as statistically significant.

**Table 1:** Brain inflammation and damage over time in mice infected with TIGR4 or FP23

**Strain**^**a**^	**Inflammation score**		***p***^**d**^	**Damage score**		***p***^**d**^
	**[median (IQR)]**^**b**^			**[median (IQR)]**^**c**^		
	**24 h**	**48 h**		**24 h**	**48 h**	
TIGR4	2 (2–6)	12 (2–12)		2 (2–3)	6 (1–8)	
			<0.001			<0.05
FP23	0 (0–0.75)	0		0 (0–3)	0 (0–1.75)	

^a^Two groups of MF1 mice were infected via the i.c. route with 10^5^ cfu/mouse of TIGR4 (n = 6) or FP23 (n = 16). Animals were sacrificed at 24 or 48 h, and brains were removed and treated for haematoxylin-eosin staining. Three (TIGR4) or 8 (FP23) mice per time-point were used.

^b^Inflammation in the brain was evaluated by counting the number of PMN in one power field (× 40) and calculating a score as follows: 0 (0 PMN), 1 (<10 PMN), 2 (10–50 PMN) 3 (>50 PMN). Four different brain regions were analysed: superficial meningeal regions over the convexities, frontal interhemispheric region, hippocampal fissure and third ventricle. For every animal, scores of each brain region were summed up into a total inflammation score (IS). Results are expressed as the median IS with the interquartile range (IQR) for each mouse group at 24 and 48 h.

^c^Neuronal damage was evaluated by estimating the percent of damaged neurons in one power field (× 40) and calculating a score as follows: 0 (no damaged neurons), 1 (<10%), 2 (10-30%), 3 (>30%). Four different brain areas were analysed: neocortex, striatum, hippocampus/dentate gyrus and cerebellum. For each mouse, scores of each brain region were summed, and the resulting number represented the total damage score (DS). Results are expressed as the median DS with the interquartile range (IQR) for each mouse group at 24 and 48 h.

^d^Mann–Whitney U test. Differences in inflammation and damage between the groups infected with TIGR4 and FP23 were analysed by combining IS and DS scores from all mice of each group, regardless of the euthanasia time.

## Results

Analysis of virulence of TIGR4 and the isogenic mutants FP28 (PspC-), FP262 (PspA-) and FP23 (rough) was performed both *in vivo* in a PM mouse model and *in vitro* using a murine microglial cell line. Although a few results on FP23 have already been published [42], we still decided to include this strain in all experiments to provide the reader with a complete comparative view of the different behaviours of pneumococcal mutants devoid of three major virulence determinants.

### Analysis of mouse survival after infection with pneumococcal strains

**Figure 1:**
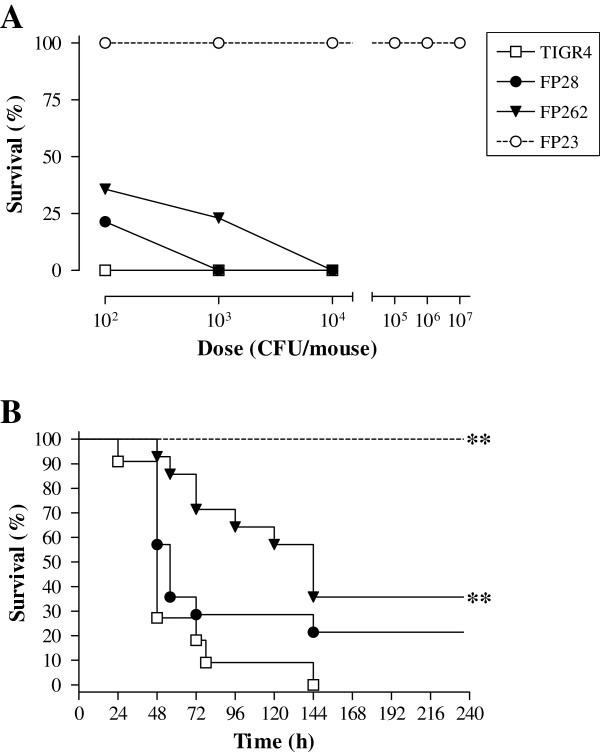
**Survival curves of mice infected with *S. pneumoniae *strains. A.** MF1 mice were infected by the i.c. route with 10^2^, 10^3^ and 10^4^ cfu/mouse of TIGR4 (wt, open squares), FP28 (PspC-, closed circles) and FP262 (PspA-, closed triangles). As a control, animals were also inoculated with different doses (10^2^-10^7^ cfu/mouse) of the unencapsulated mutant FP23 (open circles). Percent survival at different doses is shown. Data of two independent experiments are combined. **B.** Kaplan-Meyer curve of mouse survival following infection with 10^2^ cfu/mouse of TIGR4, FP28, FP262 and FP23. Mice were monitored for 10 days. Asterisks indicate statistical significance (**, *p* < 0.001; Log Rank test).

Based on previous data on lethal doses of *S. pneumoniae* killing 50% of animals (LD_50_) in experimental PM [39], mice were infected by the i.c. route with different doses of *S. pneumoniae* strains TIGR4, FP28, FP262 and FP23. Analysis of mouse clinical parameters (body temperature and weight) showed no significant differences among the groups infected with the encapsulated strains (data not shown). All animals died following challenge with 10^2^ cfu of TIGR4, while survival percentages of mice infected with FP28 and FP262 were 21 and 36, respectively (Figure 1A). At 10^3^ cfu/mouse, survival dropped to 0 and 23% in the groups infected with FP28 and FP262, respectively (Figure 1A). No animals survived challenge with 10^4^ cfu of TIGR4, FP28 and FP262 (Figure 1A). The LD_50_ of all strains, except for FP23, were below 10^2^ cfu/mouse. Upon time-to-death analysis, mice infected with the PspA-deficient strain FP262 showed significantly prolonged survival at all doses (Figure 1B and Additional file 1: Table S1, *p* < 0.01). In order to unravel differences among the groups, a Kaplan-Meyer analysis was carried out only on data from mice infected with the smallest dose of 10^2^ cfu. Median survival times of rodents challenged with TIGR4, FP28, FP262 and FP23 were 48, 56, 144 and >240 h with significant differences for the groups TIGR4-FP262 and TIGR4-FP23 (Figure 1B and Additional file 1: Table S1, *p* < 0.01 for both groups). In accordance with previously published data [42], mice injected with the rough mutant FP23 presented clinical signs comparable to uninfected naïve animals and survived all challenge doses.

### Replication of pneumococcal strains in the brain and blood of mice following i.c. infection

**Figure 2:**
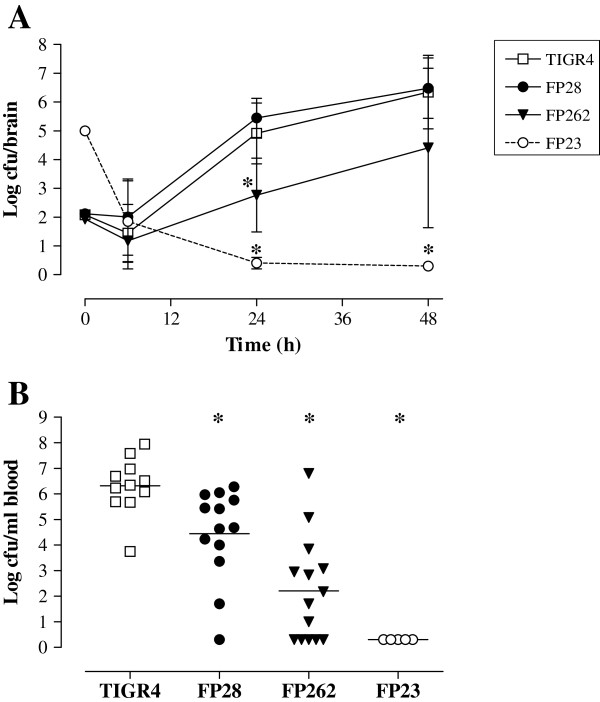
**Bacterial loads in the brain and blood of mice i.c. challenged with *S. pneumoniae*. A.** Mice were infected with 10^2^ cfu/mouse of strains TIGR4 (wt, open squares), FP28 (PspC-, closed circles) and FP262 (PspA-, closed triangles). Control mice were inoculated with 10^5^ cfu/mouse of the mutant FP23 (rough, open circles). Animals were sacrificed at different time-points (6 , 24 and 48 h) after infection, and brains were collected to determine the viable counts. Results are represented as mean log (± SD) cfu/brain over time. **B.** Animals were infected with 10^2^ cfu/mouse of strains TIGR4, FP28, FP262 and FP23. Twenty-four h after infection, blood was collected and subjected to viable counts. Data are shown as log cfu/ml of blood, and horizontal bars represent the mean cfu for each group. For both panels, asterisks indicate statistical significance (*, *p* < 0.05; Bootstrap method).

Analysis of the growth features of the mutants in standard media showed no differences compared with TIGR4 (data not shown).To evaluate their capability to replicate in the CNS, viable counts were determined over time (at 6, 24 and 48 h) in the brain of infected mice. No differences in bacterial titers were observed between FP28 and TIGR4 at any time-point. In contrast, the FP262 bacterial load in the brain was significantly lower than that of TIGR4 at 24 h post-infection (Figure 2A and Additional file 1: Table S2; *p* < 0.05). This finding suggests that the PspA mutant is impaired at replicating in the CNS in the early phase of infection. As previously reported [42], the rough strain FP23 was cleared from the brain by 24 h despite injection of animals with a high bacterial inoculum. As our PM model is characterised by the occurrence of both meningitis and sepsis [39], pneumococci were enumerated in the blood 24 h after i.c. infection with different bacterial doses (10^2^-10^4^ cfu/mouse). At the lowest inoculum of 10^2^ cfu/mouse, the number of cfu counts was significantly different between mice infected with TIGR4 and those challenged with the mutants FP28, FP262 and FP23 with a progressively decreasing trend in mean log cfu/ml of blood (Figure 2B and Additional file 1: Table S3; *p* < 0.05). In addition, significant differences could also be observed for the groups TIGR4-FP262 (at 10^3^ cfu/mouse; *p* < 0.05) and TIGR4-FP23 (at 10^3^ and 10^4^ cfu/mouse, *p* < 0.05), (Additional file 1: Table S3).

### Evaluation of PM development in mice infected i.c. with *S. pneumoniae* strains

To assess whether the mutant strains were able to induce PM, mice were infected i.c. with 10^4^ cfu of TIGR4 and the three mutants. Brains were collected 24 h after infection and subjected to histological analysis. Results showed the presence of granulocytic infiltrations involving both the subarachnoid and ventricular spaces of the brain from mice inoculated with all the encapsulated strains, and no major differences could be observed between animals infected with TIGR4 and those challenged with the mutants FP28 and FP262 (data not shown). In contrast, no inflammation was detected in the brain of animals challenged with the rough FP23 mutant.

**Figure 3:**
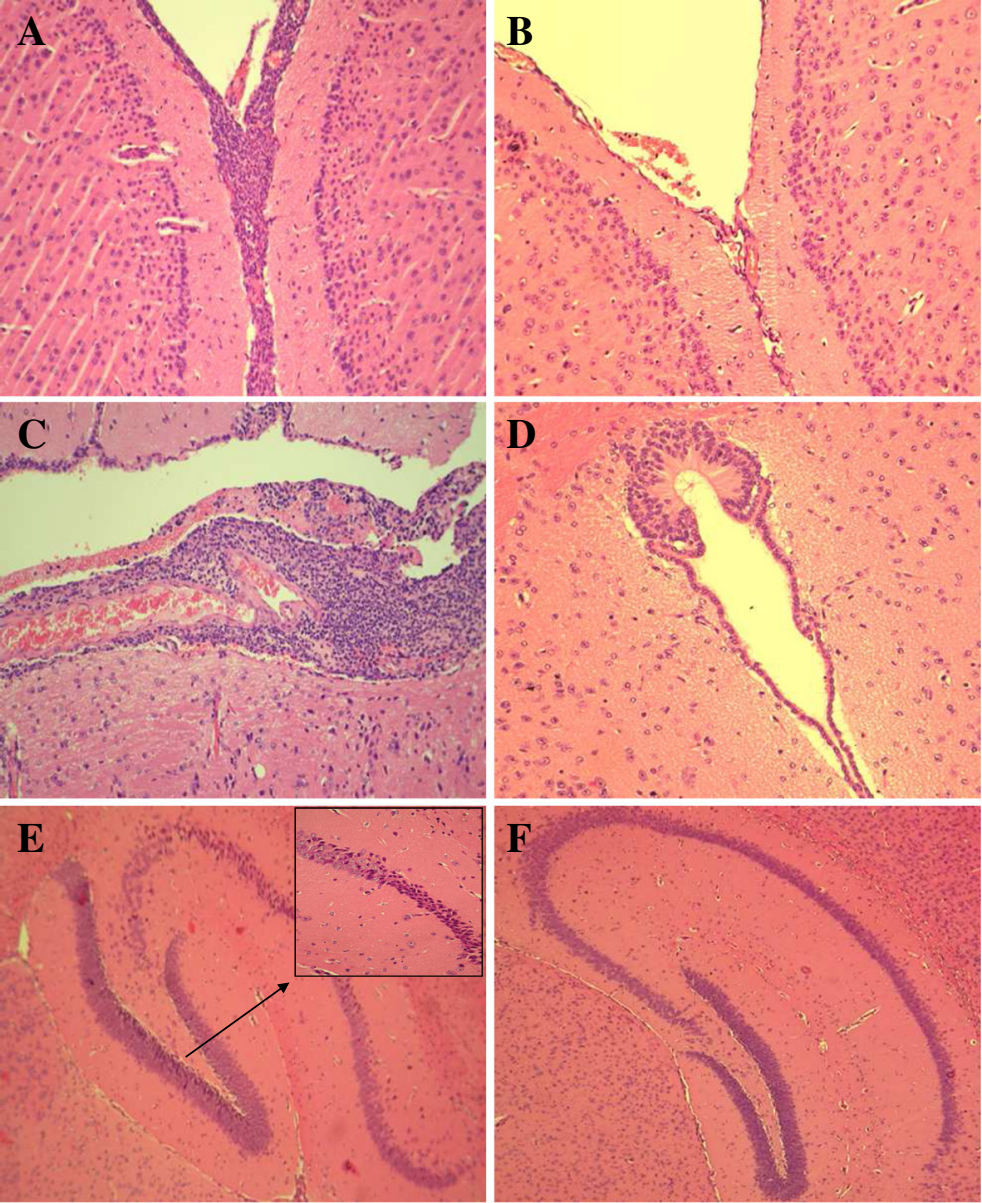
**Brain histology of mice infected with TIGR4 or FP23.** Mice were infected i.c. with 10^5^ cfu/mouse of TIGR4 or FP23 and sacrificed 24 h later. Brains were removed, fixed in formalin, embedded in paraffin, and stained with haematoxylin-eosin. The histopathological features of surface meninges **(A, B)**, ventricular spaces **(C, D)** and hippocampus **(E, F)** of animals infected with TIGR4 **(A, C, E)** or FP23 **(B, D, F)** were compared. **A, C.** Severe inflammation with massive PMN infiltrations over the inter-hemispheric fissure **(A)** and in the third ventricle **(C)**. A bulk of fibrin is clearly visible in the ventricular space **(C)**. **B, D.** No signs of inflammation on the meninges **(B)** and in a ventricle **(D)**. **E, F**. Brain damage in the dentate gyrus (neuronal shrinkage is shown in the inset) of mice infected with TIGR4 **(E)**, while the hippocampus of animals infected with FP23 was normal **(F)**.

To further investigate this evidence, two groups of mice were inoculated with 10^5^ cfu of TIGR4 and FP23 and euthanised 24 and 48 h later. Histological analysis of the brain from mice injected with TIGR4 showed severe inflammation characterised by massive infiltrations of PMN both on the meninges (Figure 3A) and in the ventricles, where accumulation of fibrin was also observed (Figure 3C). In contrast, the unencapsulated FP23 strain failed to cause inflammation on the meninges (Figure 3B) and in the ventricular spaces (Figure 3D). The TIGR4 strain also induced brain injury in the dentate gyrus of hippocampus, where shrunk neurons with picnotic nuclei were found (Figure 3E). No damage was found in samples from mice infected with FP23 (Figure 3F). A semi-quantitative analysis of brain inflammation and damage over time was carried out by determining the number of infiltrating PMN and the percentage of injured neurons in different brain regions, respectively, and by assigning each animal with an inflammation score (IS) and a damage score (DS). All mice infected with TIGR4 presented mild to severe inflammation that increased over time reaching a median IS (with IQR) of 12 (2–12) at 48 h post-infection (Table 1). Likewise, all animals challenged with TIGR4 showed neuronal injury of different degrees peaking at 48 h with median DS (IQR) of 6 (1–8) (Table 1). In contrast, median IS and DS from mice infected with the FP23 strain were equal to 0 at both time-points (Table 1), although some mice still showed mild brain damage (data not shown). The above data were also confirmed by infecting animals with a larger dose of FP23 (10^7^ cfu/mouse) and analysing their brains at a later time-point (72 h) (data not shown). The brain tissue of control mice injected with PBS appeared normal. Differences in brain inflammation (*p* < 0.001) and neuronal damage (*p* < 0.05) between the groups infected with TIGR4 and FP23 were statistically significant.

### *In vitro* interaction of *S. pneumoniae* with microglia

**Figure 4:**
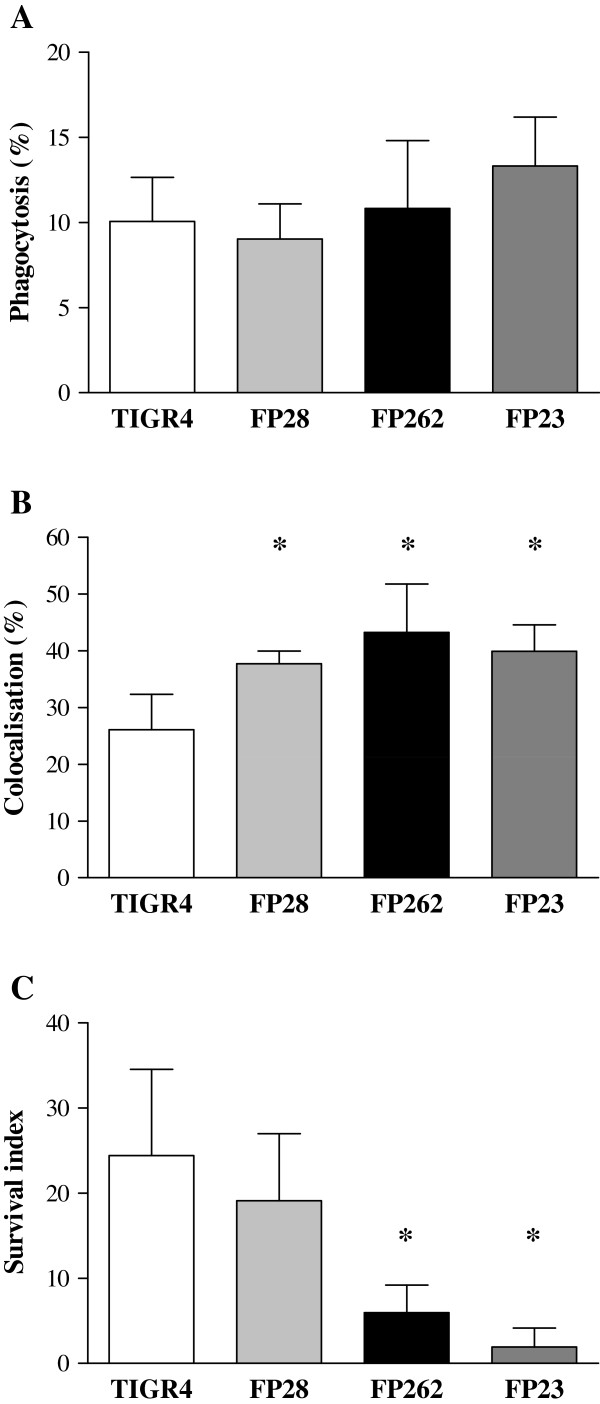
**Phagocytosis and intracellular survival of *S. pneumoniae *strains in microglial cells. A.** BV2 cells were infected for 3 h with bacteria (moi = 10). A minimum of 200 BV2 cells were examined, and any cell containing one or more bacteria was considered as phagocytic. **B.** Quantification of acidic phagosomes in microglial cells infected with *S. pneumoniae*. Hoechst-labelled bacteria were exposed to BV2 cells (moi = 10) for 3 h, and the acidotropic dye LysoTracker Red DND-99 was added. Accumulation of the dye in phagosomes containing Hoechst-labelled bacteria was observed by epifluorescence microscopy. At least 200 BV2 cells were counted, and the number of cells with bacteria-containing acidic phagosomes was scored. The percentage of colocalisation was determined as the number of BV2 cells with pneumococci-containing phagolysosomes over the total number of phagocytes. **C.** Intracellular survival of pneumococcal strains within microglial cells. BV2 cells were infected for 3 h with *S. pneumoniae* strains (moi =10), washed to eliminate extracellular bacteria, and treated with antibiotics (time 0). After 4 h, BV2 were lysed, and viable counts were performed. The SI was calculated as the number of cfu detected at time 4 h divided by the cfu number at time 0 h post-phagocytosis. For all above assays, data from 4–5 independent experiments are shown as mean ± SD. Asterisks indicate statistical significance (*, *p* < 0.05; Bootstrap method).

The susceptibility of TIGR4 and FP23 to BV2 microglial cells was recently investigated to elucidate the importance of the capsule in phagocytosis and killing by brain macrophages [42]. To understand the role of PspC and PspA in the interaction of *S. pneumoniae* with microglia, phagocytosis and intracellular survival of FP28 and FP262 were assessed in comparison with the parental strain TIGR4. The rough strain FP23 was employed as a control. By using a previously established fluorescent assay that allows to distinguish attached from internalised bacteria [42,43], the number of phagocytic cells was measured at 3 h post-infection. Levels of phagocytosis were similar for all strains (Figure 4A and Additional file 1: Table S4), suggesting that in a serotype 4 background PspC and PspA do not significantly affect uptake by BV2 microglial cells. As the TIGR4 strain was shown to resist intracellular killing by BV2 cells despite being phagocytosed to the same extent of its isogenic unencapsulated strain FP23 [42], we investigated the behaviours of FP28 and FP262 in microglial cells. Bacteria associated with acidic phagosomes (phagolysosomes) were visualised by incubating Hoechst-labelled pneumococci with BV2 cells in the presence of LysoTracker, a marker of phagosome acidification. The number of phagolysosomes within each microglial cell was determined, and the percentage of colocalisation was calculated. At 3 h post-infection, the mean percentage of TIGR4-containing phagolysosomes was significantly lower (*p* < 0.05) than those of all mutant strains, suggesting that PspC and PspA may influence phagosome maturation in microglia (Figure 4B and Additional file 1: Table S4). Finally, to examine the capability of FP28 and FP262 to survive within microglia, an intracellular survival assay was carried out by infecting BV2 cells with bacteria for 3 h, followed by treatment with antibiotics, and then counting the number of intracellular surviving pneumococci at 4 h post-phagocytosis. Consistently with previous data [42], the survival index of FP23 was significantly lower (13-folds; *p* < 0.05) than that of TIGR4. Survival of the PspA mutant FP262 was also significantly lower (4-folds; *p* < 0.05) compared with the wt strain. No differences were observed between TIGR4 and FP28 (Figure 4C and Additional file 1: Table S4).

## Discussion

Animal models of disease have significantly improved our knowledge on the interaction between *S. pneumoniae* and the host, and on the pathophysiological mechanisms involved in inflammation and brain damage during PM. Nevertheless, several issues remain to be clarified, including the role of different pneumococcal virulence factors in the disease. So far, only pneumolysin [29] and PavA [30] have been shown to contribute to PM development when tested via the i.c. route.

In this study, we used a mouse model of PM based on the inoculation of bacteria into the subarachnoid space [39] to test the pathogenicity of three different pneumococcal mutants in a type 4 background. PspA and PspC are well-known pneumococcal virulence factors [1,2], but their impact on PM has not been investigated before. Mice infected with the PspA-deficient strain FP262 showed increased survival and prolonged time-to-death for all bacterial inocula as well as lower viable counts in both blood (at the doses of 10^2^ and 10^3^ cfu/mouse) and brain (24 h post-infection) compared with TIGR4. Lower loads of FP262 in the blood may be due to decreased or delayed systemic spread of bacteria from the CNS and/or higher clearance from the bloodstream. PspA plays a key role in immune escape of *S. pneumoniae* by interfering with complement deposition/activation [6-9]. Attenuation of PspA mutants in sepsis models is well documented [6,7,45-48], and the *pspA* gene was also shown to be upregulated in experimental sepsis [49]. Therefore, reduced virulence of FP262 in our PM model may be partially caused by its impaired virulence in the bloodstream. In addition, the ability of the FP262 strain to multiply in the CNS was initially hampered, as evidenced by the kinetics of viable bacterial counts in the brain. Nonetheless, the FP262 was still able to induce inflammation in the brain. In contrast, virulence of the PspC-deficient strain FP28 was moderately reduced compared with TIGR4, and the only condition able to unveil such difference was bacterial enumeration in the blood following infection with 10^2^ cfu/mouse. Although PspC seems to have a limited impact in the PM model, our data do not exclude a possible role in crossing the BBB, an early pathogenic step bypassed by i.c. infection. It was in fact suggested that *S. pneumoniae* tropism for the CNS may be partially due to PspC (CbpA), which participates in bacterial translocation from the blood to the CSF [50], most likely by binding to the laminin receptor present on the endothelial cells of BBB [51].

Once *S. pneumoniae* enters the CNS after crossing the BBB [52], the brain resident macrophages act as key effectors of initial innate immunity, by clearing bacteria and recruiting peripheral blood cells to the site of infection [33]. To investigate the role of PspA and PspC in the interaction with microglia, *in vitro* phagocytosis assays were performed using the well-established mouse microglial cell line BV2 [41]. Bacterial uptake by BV2 cells was comparable among the strains, in accordance with previous observations on TIGR4 and FP23 [42]. Nonetheless, when the fate of the different mutants inside microglia was analysed by evaluating colocalisation of intracellular pneumococci with acidic phagosomes and bacterial survival, both the unencapsulated strain FP23 and the PspA mutant FP262 showed significantly increased association with phagolysosomes and killing compared with the wt. As previously shown for the type 4 polysaccharide capsule [42], these data suggest that PspA may also participate in pneumococcal resistance to microglial killing, possibly by interfering with phagosome maturation. In contrast, survival of the PspC mutant FP28 in BV2 cells was similar to that of TIGR4, despite an increased association with phagolysosomes. This observation seems to disagree with a previous work reporting that the lack of PspC increased the susceptibility of pneumococcal killing by microglia [53]. Such discrepancy may be explained by the different pneumococcal strains employed in the assays, a serotype 3 PspC mutant [53] and a serotype 4 PspC-deficient strain (this work), which are resistant and susceptible to phagocytosis by microglia, respectively. In summary, the data on PspA and PspC in our i.c. model (characterised by concurrent sepsis and meningitis) confirm previous reports on their key roles in experimental sepsis [6,7,36,45-48,54]. In addition, the decreased ability of FP262 of replicating in the brain at early time-points (24 h post-infection) together with its increased susceptibility to microglial killing also suggest that PspA may play a role in early stages of CNS infection by *S. pneumoniae*.

Rough strains are virtually unable to cause pneumococcal invasive disease [55], and to support this observation, fresh isolates from patients with pneumococcal infection are encapsulated [56]. However, early studies in a PM rabbit model showed that large inocula (10^7^ cfu/ml) of rough pneumococci could also be lethal, and that CSF inflammation could be induced by heat-killed unencapsulated *S. pneumoniae* (R6 strain) or their isolated cell walls, but not by heat-killed encapsulated pneumococci or their capsular polysaccharides [23,24,57]. Bacterial cell walls are potent inflammatory components, and the threshold of bioactivity of pneumococcal cell wall (PCW) corresponds to ~10^5^ cfu/ml of intact bacteria [58,59]. In our study, infection with up to 10^7^ cfu (in 50 μl) of unencapsulated FP23 bacteria (corresponding to 2×10^8^ cfu/ml of CSF) caused no meningeal inflammation or animal death ([42] and this work). Other than the PCW concentration, different factors may be responsible of the discrepancies observed, including the choice of different readouts to assess meningeal inflammation (*i.e.* cytochemical features of CSF versus histological analysis), the use of heat-inactivated versus live bacteria, and/or the type of PCW fragments released in the CSF of infected animals. As only specific motifs (*i.e.* trimeric stem peptides) of PCW are highly inflammatory [59], these structures may have not been accessible in FP23-infected mice, while being available in rabbits inoculated with heat-inactivated R6 bacteria [23]. Despite the lack of brain inflammation in mice injected with the FP23 mutant, a degree of neuronal damage was still found in the dentate gyrus of the hippocampus of some mice. As the effector mechanisms of neuronal damage in PM are both the host inflammatory response and the direct citotoxicity of bacterial components [19-21], the brain damage observed in mice infected with FP23 may be due to toxic molecules of *S. pneumoniae*, including pneumolysin and/or H_2_O_2_ in accordance to previous animal studies [21,27,28].

## Conclusions

The pathogenesis of PM is highly complex and multifactorial, and it is difficult to ascribe a precise role to a bacterial virulence factor in the disease. The results obtained with our i.c. model indicate a limited role for PspC, whereas PspA participates in PM pathogenesis possibly by interacting with microglial cells at an early phase of infection. The unencapsulated pneumococcal mutant failed to induce meningeal inflammation, brain injury and animal death even at high challenge doses, emphasising the pivotal role played by the capsule in invasive pneumococcal disease. The virulence factors evaluated in this study are either antigens of existing vaccines or strong candidates for vaccine development. The fact that they have been shown to contribute, to various degrees, to experimental PM support their use in current/future vaccine formulations against *S. pneumoniae* because vaccine efficacy may be enhanced by impairment of pneumococcal virulence. In conclusion, the data presented here may be relevant for translational research studies aimed at improving or developing effective and sustainable preventive measures against infectious diseases such as PM.

## Abbreviations

PspA: Pneumococcal surface protein A; PspC: Pneumococcal surface protein C; wt: Wild type; cfu: Colony forming units; PMN: Polymorphonuclear cells; CNS: Central nervous system; BBB: Blood-brain-barrier; CSF: Cerebrospinal fluid; PCW: Pneumococcal cell wall; IC: Intracranial; SI: Survival index; IS: Inflammation score; DS: Damage score; FCS: Fetal calf serum; PBS: Phosphate buffered saline; PFA: Paraformaldehyde; IQR: Interquartile range; CI: Confidence interval; SD: Standard deviation.

## Competing interests

The authors declare that they have no competing interests.

## Authors’ contributions

SR, co-ordination and design of the study, data analysis and interpretation, supervision of experimental work, writing of manuscript. AG, animal experiments and microbiological analysis. AP, statistical analyses. DC, animal experiments and manuscript revision. VB, animal experiments. SAT, histological analysis. BC, phagocytosis/survival experiments with microglia. EB, co-ordination of phagocytosis experiments. MRO, experimental design and critical reading of manuscript. SP, co-ordination of phagocytosis experiments, data evaluation, and manuscript revision. GP, co-ordination and design of the study, data evaluation. All authors read and approved the manuscript.

## Pre-publication history

The pre-publication history for this paper can be accessed here:

http://www.biomedcentral.com/1471-2334/13/444/prepub

## Supplementary Material

Additional file 1 Detailed data with related statistical analysis on mouse survival (Table S1), viable counts in the brain over time (Table S2), viable counts in the blood 24 h post-infection (Table S3), and phagocytosis/colocalisation/survival assays using microglial cells (Table S4). Click here for file
