# Supplementary material for: Contribution of different pneumococcal virulence factors to experimental meningitis in mice
Source: BMC Infect Dis. 2013 Sep 24;13:444. doi: 10.1186/1471-2334-13-444 (PMC3848944; doi:10.1186/1471-2334-13-444)
Supplement: Additional file 1 — Detailed data with related statistical analysis on mouse survival (Table S1), viable counts in the brain over time (Table S2), viable counts in the blood 24 h post-infection (Table S3), and phagocytosis/colocalisation/survival assays using microglial cells (Table S4). [file 1471-2334-13-444-S1.pdf]

Table S1. Median survival times (h) and 95% CI of mice infected with 10^2, 10^3 and 10^4 cfu/mouse of *S. pneumoniae* strains.

Strain/dose	10^2			10^3			10^4		
	Mice (n)	Median	95% CI	Mice (n)	Median	95% CI	Mice (n)	Median	95% CI
**TIGR4**	11	48	38.07-57.93	6	48	44.42-51.58	3	32	19.20-44.80
**FP28**	14	56	46.63-65.37	13	48	42.90-53.10	7	48	29.26-66.74
**FP262**	14	144**	115.89-172.11	13	72**	49.45-94.55	7	72**	43.89-100.11
**FP23**	3	>240**	-	3	>240**	-	3	>240**	-

Statistical significance (Log Rank test): TIGR4-FP262 and TIGR4-FP23 at all challenge doses ($p < 0.01$). For FP23, observations are censored because mice survived infection at all doses.

Table S2. Mean log cfu counts in the brain of mice 6, 24 and 48 h after infection with *S. pneumoniae* strains.

Strain/time	6			24			48		
	Mice (n)	Mean	SD	Mice (n)	Mean	SD	Mice (n)	Mean	SD
**TIGR4**	3	1.44	0.99	3	4.91	1.06	3	6.35	1.28
**FP28**	3	2	1.32	3	5.45	0.68	3	6.48	1.05
**FP262**	3	1.18	0.98	3	2.76*	1.28	3	4.40	2.77
**FP23**	3	1.85	1.42	3	0.43*	0.23	3	0.3*	0

Statistical significance (Bootstrap method): TIGR4-FP23 24 and 48 h after infection ($p < 0.05$), and TIGR4-FP262 24 h after infection ($p < 0.05$).

Table S3. Mean log cfu counts in the blood of mice 24 h after infection with 10^2, 10^3 and 10^4 cfu/mouse of *S. pneumoniae* strains.

**Strain/time**	**10^2**			**10^3**			**10^4**		
	**Mice (n)**	**Mean**	**SD**	**Mice (n)**	**Mean**	**SD**	**Mice (n)**	**Mean**	**SD**
**TIGR4**	11	6.35	1.16	6	6.97	3.26	3	8.56	4.85
**FP28**	13	4.45*	1.78	13	6.16	2.37	7	8.86	4.51
**FP262**	14	2.21*	2.03	13	4.58*	1.08	7	5.37	1.08
**FP23**	5	0.3*	0	5	0.3*	0	5	1.33*	2.52

Statistical significance (Bootstrap method): TIGR4-all mutants at 10^2 cfu/mouse ($p < 0.05$), TIGR4-FP23 and TIGR4-FP262 at 10^3 cfu/mouse ($p < 0.05$), and TIGR4-FP23 at 10^4 cfu/mouse ($p < 0.05$).

Table S4. Mean phagocytosis, colocalisation and survival index of *S. pneumoniae* strains in BV2 microglial cells.

**Strain/dose**	**Phagocytosis (%)**		**Colocalisation (%)**		**Survival index**	
	**Mean**	**SD**	**Mean**	**SD**	**Mean**	**SD**
**TIGR4**	10.06	2.58	26.10	6.22	24.42	10.12
**FP28**	9.02	2.07	37.71*	2.24	19.10	7.89
**FP262**	10.83	3.98	43.22*	8.57	5.94*	3.26
**FP23**	13.32	2.87	39.92*	4.66	1.92*	2.23

Statistical significance (Bootstrap method): TIGR4-all mutants for colocalisation with phagolysosomes ($p < 0.05$), TIGR4-FP262 and TIGR4-FP23 for intracellular survival ($p < 0.05$).
